# Hemoglobin glycation index and all-cause mortality in adults: insights from a decade-long prospective cohort study

**DOI:** 10.3389/fendo.2025.1586309

**Published:** 2025-05-29

**Authors:** Yue-Yang Zhang, Wen-Yan Li, Qin Wan

**Affiliations:** ^1^ Department of Endocrinology and Metabolism, Affiliated Hospital of Southwest Medical University, Luzhou, China; ^2^ Metabolic Vascular Disease Key Laboratory of Sichuan Province, Luzhou, China; ^3^ Sichuan Clinical Research Center for Diabetes and Metabolism, Luzhou, China; ^4^ Sichuan Clinical Research Center for Nephropathy, Luzhou, China; ^5^ Cardiovascular and Metabolic Diseases Key Laboratory of Luzhou, Luzhou, China; ^6^ Department of Endocrinology and Metabolism, The First People's Hospital of Zigong, Zigong, China

**Keywords:** hemoglobin glycation index, all-cause mortality, prospective cohort study, U-shaped correlation, risk fcator

## Abstract

**Background:**

The hemoglobin glycation index (HGI), an indicator of individual differences in glucose metabolism. This study undertakes a detailed 10-year cohort analysis to investigate the potential association between HGI and all-cause mortality in a Chinese adult population.

**Methods:**

Baseline data encompassing lifestyle and metabolic parameters were collected from 10,008 participants, with a subsequent 10-year follow-up. Following exclusions based on predefined criteria, 9,084 individuals were included in the final analysis. Participants were categorized into quartiles based on their HGI values. A suite of statistical tools, including Kaplan-Meier survival analysis, Cox proportional hazards models, restricted cubic splines (RCS), threshold effect models, and subgroup analyses, was employed to investigate the association between HGI and all-cause mortality.

**Results:**

During the 10-year follow-up period, a total of 514 all-cause mortality cases were recorded. Kaplan-Meier survival analysis identified the Q2 group as having the lowest mortality rate. Fully adjusted Cox proportional hazards models demonstrated significant associations, indicating higher all-cause mortality risks in participants with both extremely low and high HGI levels compared to the Q2 group. RCS analysis further illustrated a U-shaped relationship between HGI and all-cause mortality.

**Conclusions:**

In the Chinese population, both markedly elevated and significantly reduced HGI levels are associated with adverse impacts on long-term survival.

**Core tip:**

The aim of this study was to assess the association of Hemoglobin Glycation Index(HGI) with all-cause mortality in non-type 2 diabetic patients based on a 10-year cohort study from China. After COX regression, restricted cubic spline analysis, and subgroup analyses, it was found that a significant increase or decrease in HGI adversely affected long-term survival.

## Introduction

Diabetes is one of the most prevalent and severe endocrine and metabolic disorders, primarily characterized by elevated blood glucose levels resulting from absolute or relative insulin deficiency, leading to multi-organ damage and, ultimately, death (1, 2). The 2021 Global Burden of Disease, Injuries, and Risk Factors Report identified diabetes as the eighth leading risk factor for both mortality and disability (3). According to the 10th edition of the Diabetes Atlas by the International Diabetes Federation (IDF), approximately 537 million individuals worldwide are currently living with diabetes, with the number projected to increase to 653 million by 2030 and a staggering 783 million by 2045 (4). Furthermore, global healthcare expenditures related to diabetes are anticipated to exceed 1.05 trillion USD (5). These statistics highlight the profound impact of diabetes on global public health and emphasize the urgent need for more effective diabetes prevention and management strategies in the general population.

According to the 2024 diabetes guidelines published by the American Diabetes Association (ADA), glycated haemoglobin(HbA1c) measurement remains the gold standard for diagnosing diabetes, reflecting an individual’s average blood glucose over the preceding three months and serving as a key indicator for assessing the effectiveness of glycemic interventions (6, 7). However, studies have shown that only 60-80% of the variations in average blood glucose levels can be explained by changes in HbA1c levels (8). The metabolism of red blood cells, the glucose gradient across the red blood cell membrane, and genetic factors all influence HbA1c levels, independent of average blood glucose levels (9–11). Therefore, HbA1c does not fully capture the body’s glucose metabolism state.

The Hemoglobin Glycation Index (HGI), proposed by Hempe et al. (12) quantifies the association between HbA1c and average blood glucose levels. It is defined as the difference between the measured HbA1c and the predicted HbA1c, derived from a linear regression based on fasting blood glucose levels. Initially, HGI proved useful in evaluating HbA1c differences in children with type 1 diabetes (13). More recent studies suggest that HGI is a strong predictor of cardiovascular disease outcomes, with patients with high HGI showing significantly higher mortality from acute coronary syndrome and vascular dysfunction (14). Additionally, higher HGI is associated with an elevated risk of diabetes complications, including mortality and both macrovascular and microvascular issues (15). However, recent studies indicate that patients with low HGI levels may also experience more cardiovascular events than those with moderate HGI levels (16, 17). A study utilizing the MIMIC-IV database also found a strong association between HGI and all-cause mortality in critically ill coronary artery disease patients, particularly in those with low HGI (18). However, the relationship between HGI and all-cause mortality in the general population, particularly in the Chinese population, remains unclear. Therefore, this study aims to explore the potential association between HGI and all-cause mortality through a 10-year follow-up cohort from the China Cardiometabolic Disease and Cancer Cohort Study(4C Study).

## Method

### Study design and population

The 4C Study is a nationwide, multicenter, population-based prospective cohort study aimed at exploring potential associations between various metabolic factors and specific clinical outcomes, particularly diabetes and cardiovascular diseases. The study protocol and informed consent were approved by the Human Research Ethics Committee at Ruijin Hospital, Shanghai Jiao Tong University, ensuring ethical compliance. All participants provided written informed consent before participating in the study (19).

The 4C Study encompasses 20 community research sites across 16 provinces, autonomous regions, and municipalities in mainland China. Initially, eligible men and women aged 40 years and older were identified through the resident registration systems at each research site. Trained community health workers then conducted home visits to invite eligible individuals to participate in the study and follow-up. Baseline assessments for 10,008 participants were conducted between 2010 and 2011, including face-to-face interviews, baseline questionnaires, physical exams, standard oral glucose tolerance tests (OGTT), and blood sample collection. Follow-up surveys were conducted in 2014, 2016, and 2021, with 9,126 participants retained, resulting in a follow-up rate of 91.19%.

**Supplementary Figure 1** presents the flowchart outlining the inclusion and exclusion criteria for study participants. Initially, 13 participants were excluded due to missing baseline data in more than 10% of cases. Subsequently, 12 participants were excluded due to a prior diagnosis of type 2 diabetes, and 17 participants were lost to follow-up in 2021. Ultimately, the final analysis included 9,084 participants.

### Data collection

Baseline data were collected at community health stations, with standardized collection times scheduled in the morning. Participants were instructed to fast for at least 10 hours prior to their appointments. Trained health workers administered standardized questionnaires to gather detailed data on demographics, dietary habits, lifestyle factors, current and past medical histories, and medication use. Nurses, adhering to standardized protocols, measured participants’ weight, height, blood pressure, and waist circumference. Participants were instructed to rest quietly for at least 30 minutes before the measurements and to avoid alcohol, smoking, tea consumption, and vigorous exercise. Blood pressure was recorded using a standardized electronic sphygmomanometer (HEM-752 FUZZY; Omron, Dalian, China), with three readings taken and the average value calculated for accuracy. Blood samples were then collected and sent to a central laboratory for analysis of HbA1c levels and circulating metabolites, including blood lipids. HbA1c levels were quantified using high-performance liquid chromatography (VARIANT II system; Bio-Rad, Hercules, California), and blood lipids were measured using an automated analyzer (Abbott Laboratories, Abbott Park, Illinois). Smoking status was categorized as current smokers (defined as smoking at least 7 cigarettes per week for at least 6 months) and non-smokers. Alcohol use was classified into current drinkers (defined as consuming alcohol at least once a week for at least 6 months) and former drinkers (20).

### Ascertainment of covariates

Consistent with previous studies, we included a range of potential confounding variables in the baseline statistical analysis (21). The variables included demographic factors such as age and gender (classified as “male” and “female”), educational level (categorized as “below high school” and “high school and above”), and lifestyle factors such as smoking status (“yes” or “no”) and alcohol consumption status (“yes” or “no”). Furthermore, we adjusted for several clinical indicators, including systolic blood pressure (SBP), diastolic blood pressure (DBP), body mass index (BMI), fasting blood glucose (FBG), triglycerides (TG), serum creatinine (Cr), total cholesterol (TC), high-density lipoprotein cholesterol (HDL-C), low-density lipoprotein cholesterol (LDL-C), and HbA1c.

### Assessment of all-cause mortality

To confirm the outcome events for participants, we integrated data from hospital databases, national insurance system medical records, and resident records, ensuring comprehensive collection of the necessary information. All-cause mortality was defined as death from any cause, verified by cross-checking relevant documents with two independent healthcare workers.

### HGI calculation

A linear regression model was developed to examine the relationship between FBG and HbA1c using data from all participants in this study (**Supplementary Figure 2**). The X-axis represents FBG levels, while the Y-axis represents HbA1c levels. The regression equation used to predict HbA1c levels is HbA1c = 0.03 × FBG (mg/dL) + 2.95 (r^2^ = 0.68; P < 0.001). Subsequently, the observed HbA1c level was subtracted from the predicted HbA1c level to calculate each participant’s HGI(HGI = observed HbA1c - predicted HbA1c).

### Statistical analysis

The study population was categorized into four groups based on HGI quartiles: Q1 (n = 2260, HGI < -0.31), Q2 (n = 2281, -0.31 ≤ HGI < -0.01), Q3 (n = 2270, -0.01 ≤ HGI < 0.29), and Q4 (n = 2273, HGI ≥ 0.29). Baseline characteristics of participants were systematically summarized using appropriate statistical methods according to the data type. Continuous variables were expressed as mean ± standard deviation, while categorical variables were reported as counts (percentages). For normally distributed variables, analysis of variance (ANOVA) was applied; for skewed variables, the Kruskal-Wallis test was employed; and for categorical variables, the chi-square test was used. Kaplan-Meier survival analysis and log-rank tests, stratified by HGI quartiles, were performed to assess group differences. Cox proportional hazards regression was utilized to examine the potential association between HGI and all-cause mortality. Hazard ratios (HR) and 95% confidence intervals (CI) were computed using four distinct adjustment models. Model 1 was the unadjusted model; Model 2 adjusted for age and sex; Model 3 adjusted for age, sex, DBP, SBP, LDL-C, TC, Cr, FBG, and HbA1c; and Model 4 was fully adjusted for age, sex, DBP, SBP, LDL-C, TC, Cr, FBG, HbA1c, smoking status, alcohol consumption, and education level. The trend p-value was computed by treating HGI quartiles as an ordinal variable. Additionally, restricted cubic splines (RCS) with four nodes were used in Model 4 to examine the nonlinear relationship between HGI and all-cause mortality, and a threshold effect model was applied to identify the inflection point of HGI (22). Finally, subgroup analyses were conducted based on age (<60, ≥60), sex (male, female), and BMI (<25, ≥25), and interaction effects were evaluated. Non-significant interaction p-values were considered to indicate consistency across subgroups.

All statistical analyses were performed using SPSS version 26.0 and R version 4.3.3, with forest plots generated using GraphPad Prism version 10.0. Two-sided P-values < 0.05 were considered statistically significant.

## Result

### Baseline characteristics

This study included 9084 participants, with a mean age of 58.49 ± 10.01 years, consisting of 3085 men (34.0%) and 5999 women (66%). Baseline data stratified by HGI quartiles are presented in **Table 1**. The high HGI group exhibited higher age, LDL, TG, and HbA1c levels, along with lower HDL compared to the low HGI group. Participants in the Q1 and Q4 groups demonstrated significantly higher SBP, TC, and FBG levels compared to those in the Q2 and Q3 groups. Additionally, a higher proportion of participants in the Q2 group had completed high school education or higher.

**Table 1:** Baseline characteristics of participants by HGI quartile.

Variables	Tatal	Q1(<-0.31)	Q2(-0.31≤HGI<-0.01)	Q3(-0.01≤HGI<0.29)	Q4(>0.29)	P
N	9084	2260	2281	2270	2273	
Age, years	58.49 ± 10.01	56.74 ± 10.09	57.56 ± 10.11	58.73 ± 9.87	60.92 ± 9.47	<0.01
Male (%)	3085 (34.0)	782 (34.6)	744 (32.7)	773 (34.1)	786 (34.7)	0.45
BMI, kg/m^2^	23.91 ± 3.35	23.90 ± 3.19	23.92 ± 3.36	23.77 ± 3.35	24.03 ± 3.50	0.14
DBP, mmHg	77.66 ± 11.54	78.07 ± 11.69	77.75 ± 11.50	77.46 ± 11.55	77.36 ± 11.43	0.17
SBP, mmHg	127.41 ± 20.91	128.19 ± 20.77	126.56 ± 20.97	126.58 ± 20.81	128.41 ± 21.05	<0.01
LDL-c, mmol/L	2.59 ± 0.82	2.51 ± 0.81	2.59 ± 0.81	2.61 ± 0.80	2.64 ± 0.87	<0.01
HDL-c, mmol/L	1.24 ± 0.34	1.27 ± 0.35	1.27 ± 0.35	1.23 ± 0.33	1.21 ± 0.34	<0.01
TC, mmol/L	1.62 ± 1.28	1.70 ± 1.47	1.54 ± 1.22	1.58 ± 1.17	1.67 ± 1.22	<0.01
TG, mmol/L	4.60 ± 1.14	4.56 ± 1.13	4.61 ± 1.12	4.60 ± 1.10	4.61 ± 1.22	<0.01
Cr, mmol/L	65.10 ± 21.82	66.23 ± 18.45	65.10 ± 25.75	64.16 ± 17.26	64.95 ± 24.49	0.02
FBG, mmol/L	5.92 ± 1.68	6.29 ± 1.88	5.67 ± 1.18	5.57 ± 1.15	6.15 ± 2.16	<0.01
HbA1c, %	6.16 ± 1.10	5.67 ± 0.90	5.86 ± 0.64	6.10 ± 0.63	6.99 ± 1.50	<0.01
Current smoker	1304 (14.4)	316 (14.0)	298 (13.1)	340 (15.0)	350 (15.4)	0.08
Current drinker	2516 (27.7)	698 (30.9)	676 (29.6)	623 (27.4)	519 (22.8)	<0.01
Senior high school and above	2084 (22.9)	669 (29.6)	727 (31.9)	691 (30.4)	600 (26.4)	<0.01

Data are summarized as number (percentage), mean ± standard deviation. HGI, hemoglobin glycation index; BMI, body mass index; DBP, diastolic blood pressure; SBP, systolic blood pressure; LDL-C, low-density lipoprotein cholesterol; HDL-C, high-density lipoprotein cholesterol; TC, total cholesterol; TG, triglyceride; Cr, creatinine; FBG, fasting blood glucose; HbA1c, glycated haemoglobin.

### Survival analysis

**Figure 1:**
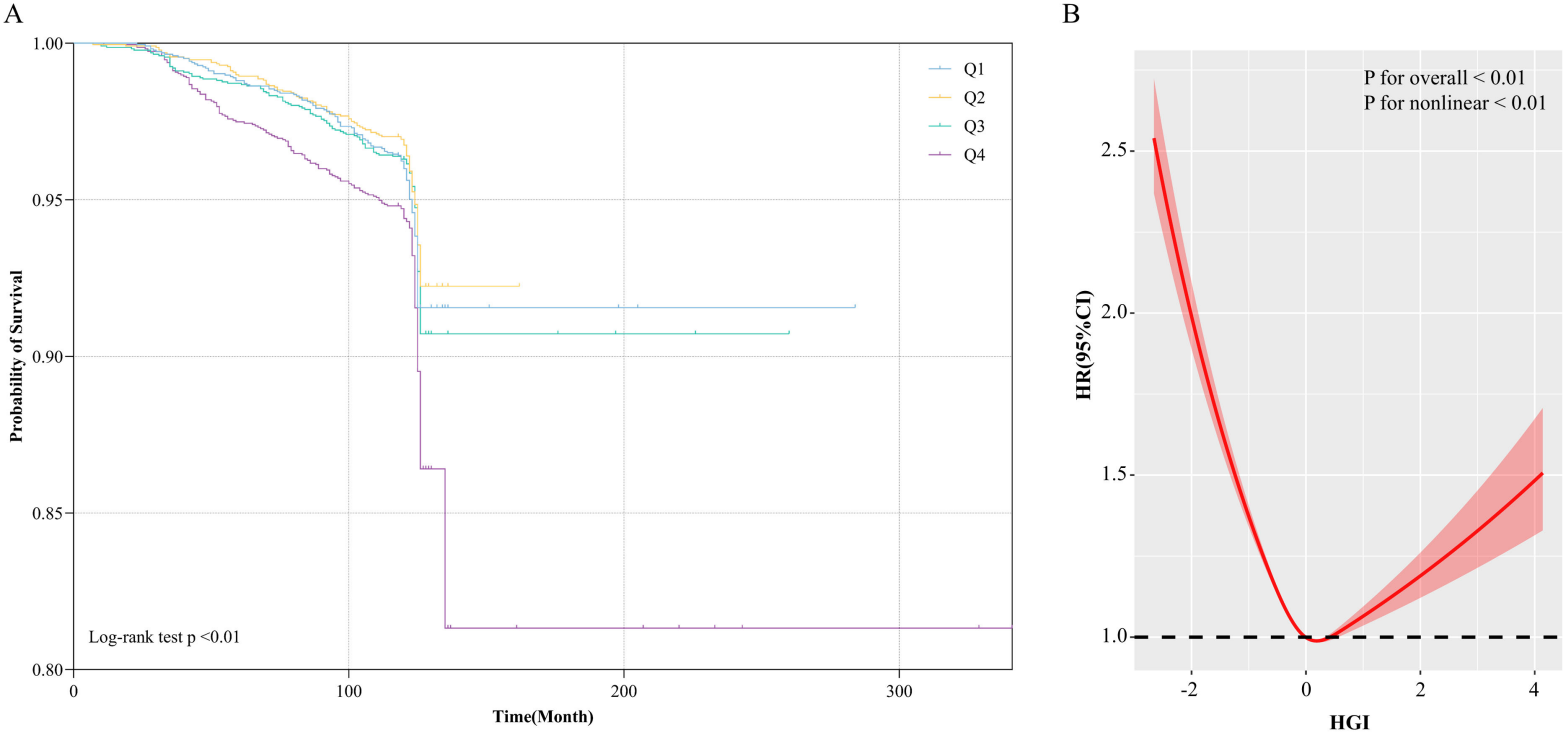
**(A)** Kaplan-Meier all-cause mortality survival analysis curve; **(B)** Results of RCS analysis of the association between HGI and all-cause mortality. Adjusted for age, sex, DBP, SBP, LDL-c, HDL-c, TG, TC, Cr, FBG, HbA1c, smoking status, alcohol consumption, and education level.

Kaplan-Meier survival curves stratified by HGI quartiles were utilized to compare the incidence of all-cause mortality across the HGI groups (**Figure 1A**). The Q2 group exhibited significantly lower all-cause mortality compared to the other groups, while the Q4 group demonstrated the highest all-cause mortality (Log-rank test p < 0.01). This suggests that both high and low HGI levels may negatively impact long-term survival, with particularly detrimental effects associated with high HGI.

### Association of HGI with all-cause mortality

The survival analysis revealed that the Q2 group exhibited the lowest all-cause mortality in comparison to the other groups. Building upon these findings, the Q2 group was used as the reference to construct a Cox proportional hazards model for assessing the potential association between HGI and all-cause mortality. The results indicated that, during the 10-year follow-up, a total of 514 all-cause deaths were recorded, corresponding to an overall mortality rate of 5.66%. The Q4 group exhibited the highest all-cause mortality rate (7.74%), followed by the Q1 group (5.22%). In the fully adjusted model (Model 4), both low and high HGI levels were significantly associated with all-cause mortality compared to the Q2 group (Q1 vs Q2: HR, 1.32 [1.04, 1.73], p < 0.05; Q4 vs Q2: HR, 1.32 [1.12, 1.53], p < 0.05). Therefore, we conclude that both high and low HGI levels represent significant risk factors for all-cause mortality (**Table 2**). Subsequently, we employed RCS modeling, which revealed a “U”-shaped relationship between HGI and all-cause mortality risk (p for nonlinear < 0.01, **Figure 1B**). The threshold effect analysis identified an HGI threshold of 0.07 for all-cause mortality (**Table 3**).

**Table 2:** Hazard ratios and 95% CIs for the association of HGI with all-cause mortality.

Variables	Q1	Q2	Q3	Q4	P	P for trend
Total number of events/total number of persons(Incident rate)				514/9084(5.66%)		
Event/total	118/2260	104/2281	116/2270	176/2273		
Incident rate (%)	5.22%	4.56%	5.11%	7.74%		
Model 1	1.42(1.14,1.79)	Reference	1.07(0.82,1.40)	1.62(1.27,2.07)	0.01	0.01
Model 2	1.39(1.09,1.77)	Reference	0.95(0.73,1.24)	1.58(1.29,1.91)	0.02	0.80
Model 3	1.35(1.05,1.78)	Reference	0.95(0.72,1.25)	1.50(1.22,1.69)	0.02	0.43
Model 4	1.32(1.04,1.73)	Reference	1.04(0.78,1.39)	1.32(1.12,1.53)	0.02	0.58

Model 1: unadjusted model;

Model 2: adjusted for age and sex;

Model 3: adjusted for age, sex, DBP, SBP,HDL, LDL-C, TC, TG,Cr, FBG, HbA1c;

Model 4: adjusted for age, sex, DBP, SBP,HDL, LDL-C, TC, TG,Cr, FBG, HbA1c, smoking status, alcohol consumption, and education level.

**Table 3:** Threshold effect analysis.

Variables	All-cause mortality
Standard linear regression	0.88 (0.77-1.02), P = 0.09
Inflection point	0.07
<0.07	0.73 (0.58-0.91), P =0.01
>0.07	1.31 (1.11-1.65), P =0.03
P for likelihood ratio test	0.01

### Subgroup analyses

**Figure 2:**
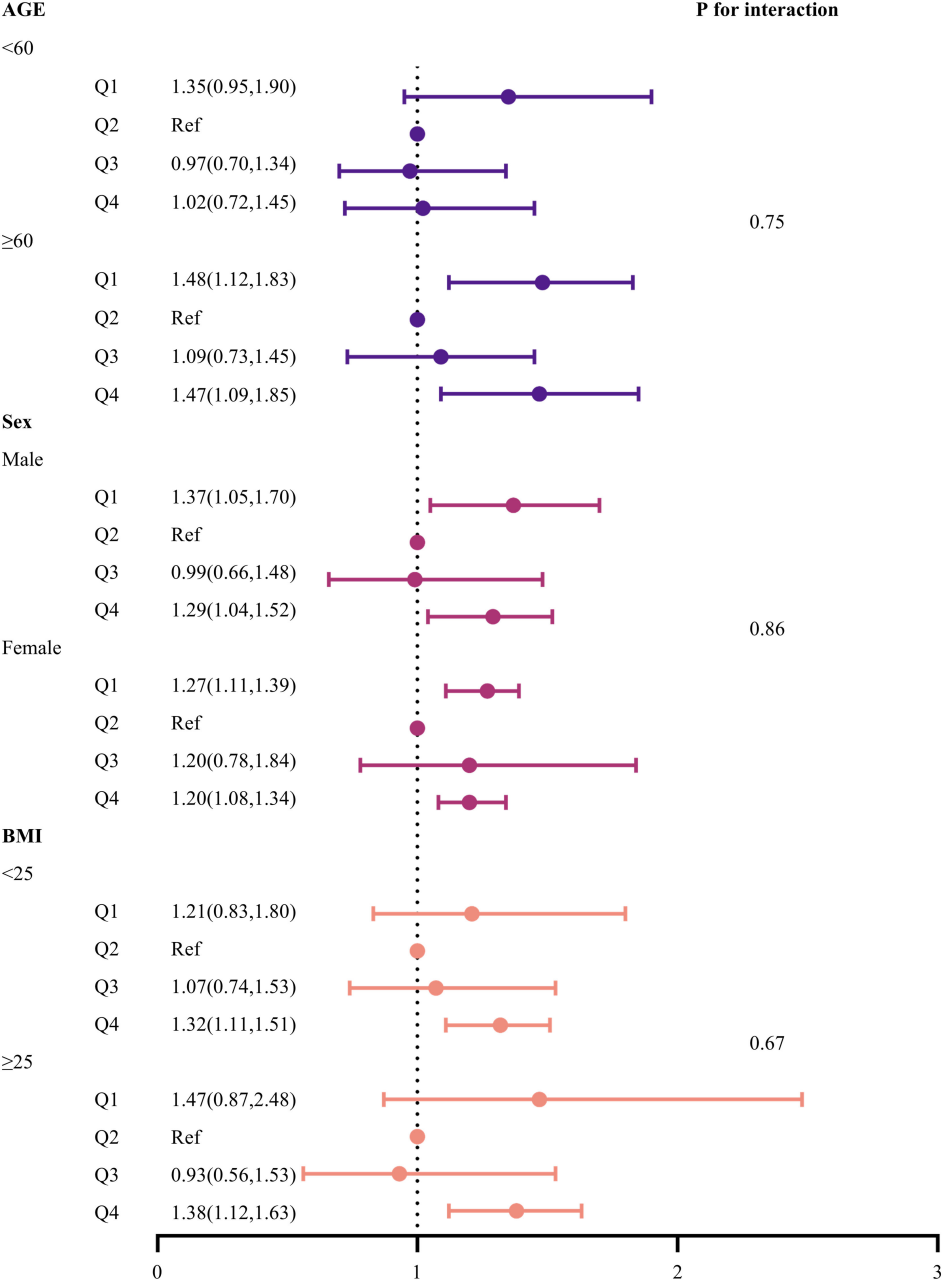
Forest plot of subgroup analyses of the relationship between HGI and all-cause mortality. Adjusted for age, sex, DBP, SBP, LDL-c, HDL-c, TG, TC, Cr, FBG, HbA1c, smoking status, alcohol consumption, and education level.

Finally, a risk subgroup analysis was performed based on age, gender, and BMI (**Figure 2**). The results indicated that both low (Q1) and high (Q4) HGI levels were significantly associated with all-cause mortality in the subgroups of individuals aged ≥ 60 years, males, and females. Additionally, high HGI (Q4) was strongly associated with all-cause mortality across all BMI subgroups. Furthermore, no significant interactions were observed between HGI levels and subgroup variables (p for interaction > 0.05).

## Discussion

In this retrospective cohort study of 9,084 Chinese adults, we found that both high and low HGI levels were significantly associated with all-cause mortality when compared to individuals with moderate HGI levels. The RCS-based analysis revealed a distinct U-shaped relationship between HGI and the risk of all-cause mortality, with a threshold value of 0.07, suggesting that both high and low HGI levels may impact long-term survival to varying degrees, consistent with the findings from the Cox proportional hazards model. Therefore, these findings suggest that HGI could serve as an independent risk factor for all-cause mortality and has the potential to function as an early biomarker for monitoring an individual’s survival status.

HbA1c is formed through a non-enzymatic reaction between intracellular HbA1 and glucose, leading to discrepancies between actual and predicted HbA1c levels, particularly among different individuals, though the underlying mechanism remains poorly understood (23). Since the introduction of HGI as a tool to quantify blood glucose variability across diverse populations, numerous studies have sought to explore its potential clinical utility. For example, HGI has been strongly associated with contrast-induced acute kidney injury, non-alcoholic fatty liver disease, and cardiovascular mortality (24, 25). In populations with glucose intolerance, elevated HGI levels have been associated with telomere attrition, suggesting an underlying state of inflammation and oxidative stress (26). These studies suggest that HGI may serve as an effective biomarker for monitoring an individual’s disease status.

In the Diabetes Control and Complications Trial, researchers initially found that increasing HGI was associated with a threefold and sixfold increase in the risk of retinal and kidney complications, respectively, in diabetic patients (27). However, this does not suggest that low HGI acts as a protective factor against disease, particularly concerning cardiovascular disease and mortality risk (28). FBG, a primary indicator of glucose metabolism, is similarly strongly associated with individual mortality. A bidirectional cohort study demonstrated that elevated FBG is a risk factor for 1-year all-cause mortality in COVID-19 hospitalized patients (29). In patients with type 2 diabetes, elevated FBG levels are also considered an independent risk factor for accelerating premature mortality (30). However, the relationship between FBG, HbA1c, and disease outcomes appears to be variable. This highlights the importance of detecting HGI. A study using the MIMIC-IV database, however, found that HGI exhibited a U-shaped relationship with mortality in critically ill CAD patients, where both low and high HGI were identified as risk factors for death in this population (18). In recent years, low HGI has garnered increasing attention in research. Østergaard et al. (31) suggested that low HGI may serve as a risk factor for myocardial infarction in diabetic patients. Wang et al. (32) identified a U-shaped relationship between HGI and 5-year major cardiovascular events. We hypothesize that HGI may influence individual mortality risk through the following mechanisms: First, a study employing skin endogenous fluorescence technology found that skin advanced glycation end-products are associated with HGI (33). Advanced glycation end-products are a class of heterogeneous compounds that promote low-density lipoprotein modification, induce oxidative stress, activate Toll-like receptor 4-mediated pro-inflammatory signaling, and drive fibrosis and endothelial dysfunction, all of which are implicated in the pathophysiology of diabetic complications, aging, and Alzheimer’s disease (34–36). Second, advanced glycation end-products contribute to insulin resistance and β-cell dysfunction, leading to glucose metabolism disorders (37). This study found that both low and high HGI are associated with metabolic disorders, including lipid, glucose, and protein metabolism, all of which negatively affect long-term survival.

### Strengths and limitations

This study offers several notable advantages. First, the study involved a representative Chinese population with a substantial sample size. Secondly, this cohort study benefits from an extended follow-up period and high response rates, providing a robust framework that mitigates the inherent biases of cross-sectional studies and enhances the reliability of the findings. Third, the 4C study was carried out by trained healthcare professionals adhering to standardized protocols for data collection and disease diagnosis, significantly reducing potential bias due to human factors. Finally, the use of RCS, threshold effect models, and subgroup analyses bolstered statistical power and affirmed the robustness of the results.

Despite its strengths, this study has several limitations. First, unaccounted variables, such as dietary patterns, racial differences, and lifestyle factors, may also affect the results. Secondly, as the study primarily involved participants from China, caution is warranted when generalizing the findings to other populations. Third, the calculation of HGI relies on data from a specific population, requiring the construction of a new regression equation for each study. Fourth, the inherent limitations of observational studies preclude establishing clear causal relationships. Fifth, the lack of data on the specific causes of death limited our ability to conduct a more nuanced analysis of the association between HGI and mortality. Future follow-up studies are planned to address this gap and provide deeper insights.

## Conclusions

In conclusion, within the Chinese population, HGI is U-shapedly associated with the risk of all-cause mortality, with both elevated and reduced HGI levels serving as risk factors influencing long-term survival.

## Data Availability

The raw data supporting the conclusions of this article will be made available by the authors, without undue reservation.

## Supplementary material

Supplementary Figure 1 Flowchart for the Selection of the Analyzed Study Sample From the 4C study’s database

Supplementary Figure 2 Linear correlation between the FBG and HbA1c levels. Predicted HbA1c level = 0.03 × FBG (mg/dL) + 2.95 as revealed by linear regression (black solid line). FBG, Fasting blood glucose; HbA1c, Glycosylated hemoglobin.
