# Supplementary figures and images for: Hemoglobin glycation index and all-cause mortality in adults: insights from a decade-long prospective cohort study
Source: Front Endocrinol (Lausanne). 2025 May 29;16:1586309. doi: 10.3389/fendo.2025.1586309 (PMC12158740; doi:10.3389/fendo.2025.1586309)

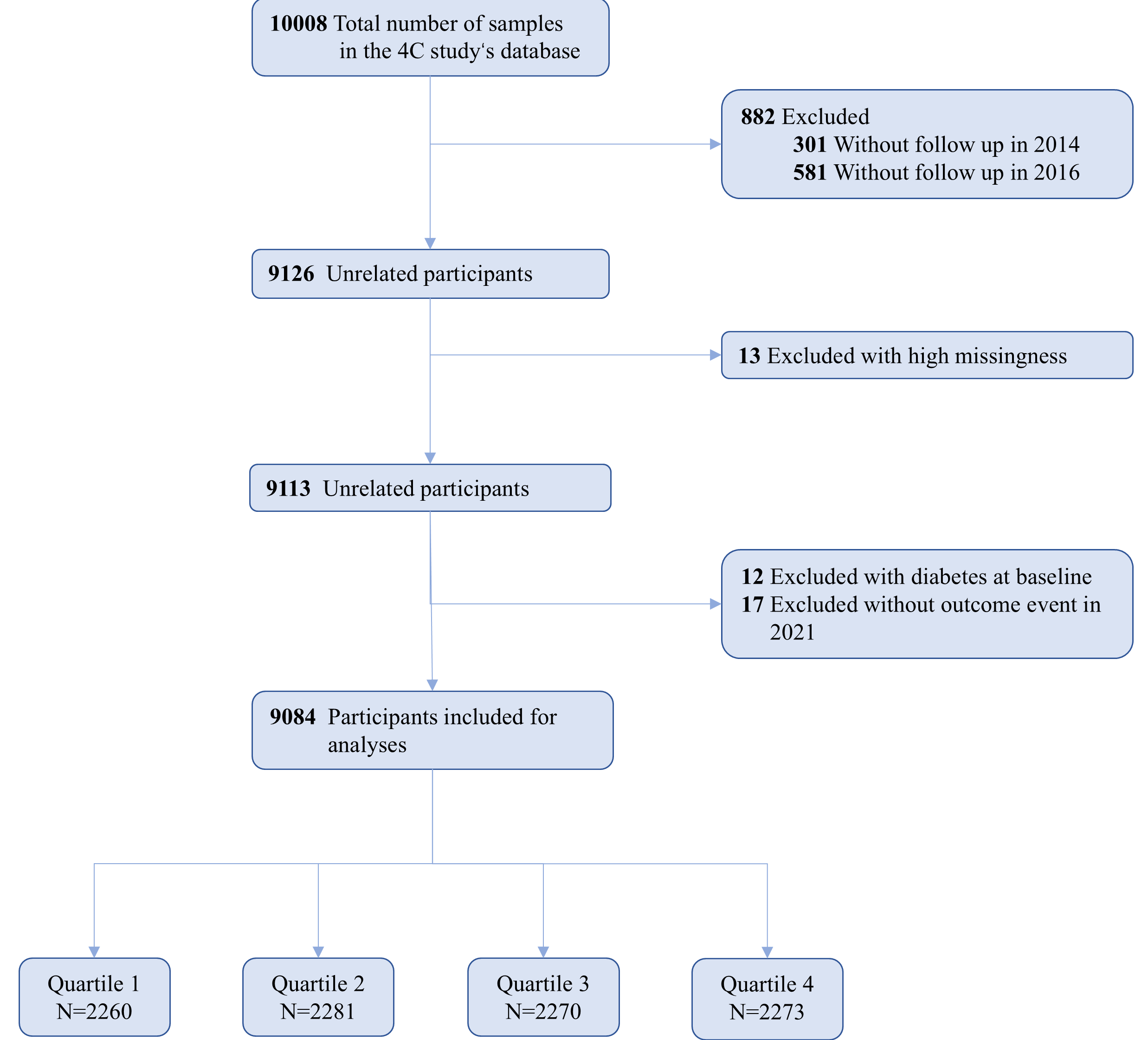

Supplement: Supplementary Figure 1 — Flowchart for the Selection of the Analyzed Study Sample From the 4C study’s database [file Image1.tif]

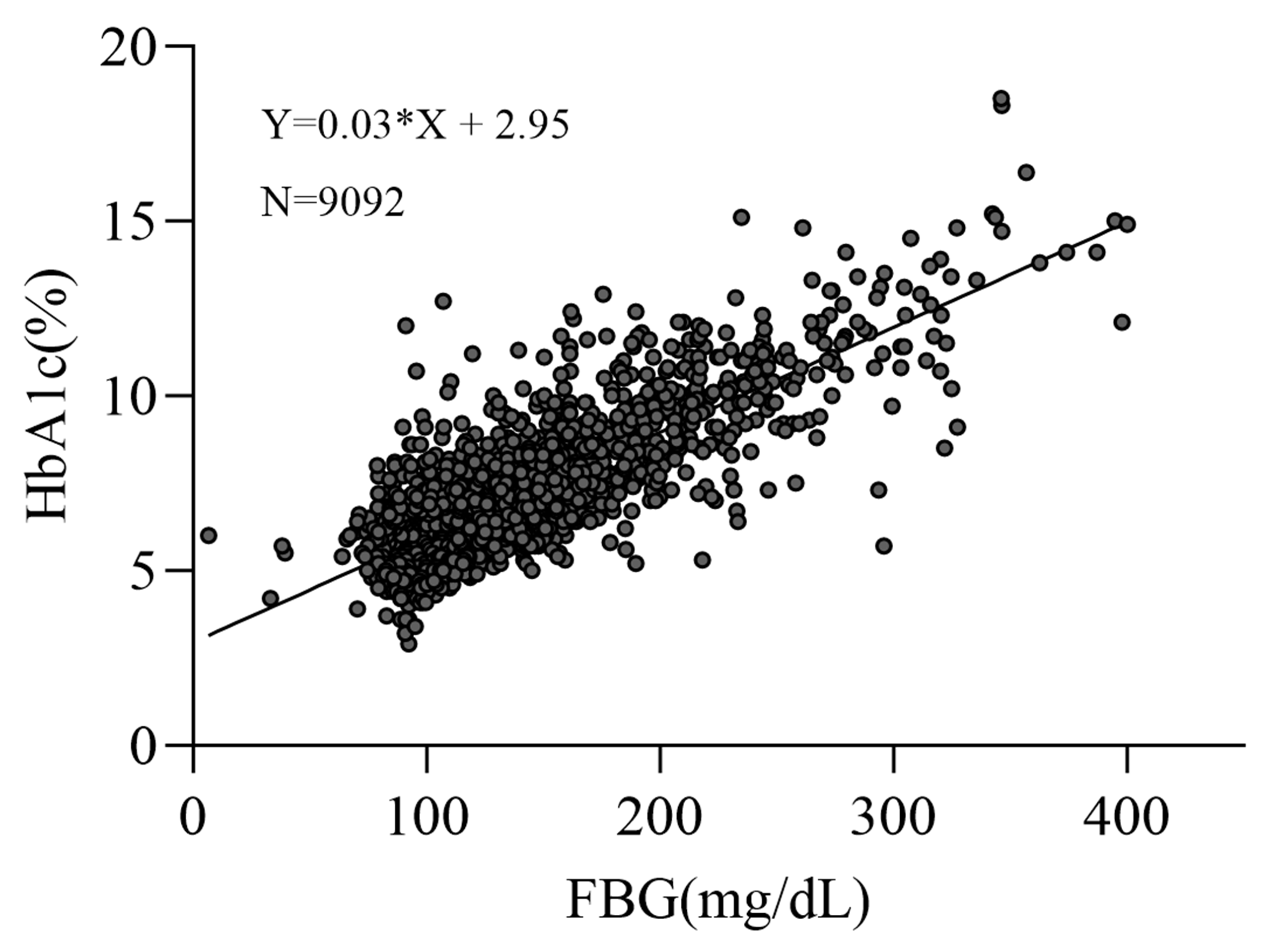

Supplement: Supplementary Figure 2 — Linear correlation between the FBG and HbA1c levels. Predicted HbA1c level = 0.03 × FBG (mg/dL) + 2.95 as revealed by linear regression (black solid line). FBG, Fasting blood glucose; HbA1c, Glycosylated hemoglobin. [file Image2.tif]
